# Use of semantic segmentation for mapping *Sargassum* on beaches

**DOI:** 10.7717/peerj.13537

**Published:** 2022-06-09

**Authors:** Javier Arellano-Verdejo, Martin Santos-Romero, Hugo E. Lazcano-Hernandez

**Affiliations:** 1 Department of Observation and Study of the Earth, Atmosphere and Ocean, El Colegio de la Frontera Sur, Chetumal, Quintana Roo, Mexico; 2 Universidad Da Vinci, Mexico, Ciudad de Mexico, Mexico; 3 Department of Observation and Study of the Earth, Atmosphere and Ocean, CONACYT-El Colegio de la Frontera Sur, Chetumal, Quintana Roo, Mexico

**Keywords:** Deep learning, Beach monitoring, Ecological application, Citizen science, Sargasso

## Abstract

The unusual arrival of *Sargassum* on Caribbean beaches is an emerging problem that has generated numerous challenges. The monitoring, visualization, and estimation of *Sargassum* coverage on the beaches remain a constant complication. This study proposes a new mapping methodology to estimate *Sargassum* coverage on the beaches. Semantic segmentation of geotagged photographs allows the generation of accurate maps showing the percent coverage of *Sargassum*. The first dataset of segmented *Sargassum* images was built for this study and used to train the proposed model. The results demonstrate that the currently proposed method has an accuracy of 91%, improving on the results reported in the state-of-the-art method where data was also collected through a crowdsourcing scheme, in which only information on the presence and absence of *Sargassum* is displayed.

## Introduction

The macroalgae *Sargassum* spp. belongs to the class Phaeophyta(brown algae) that inhabit the seas worldwide. Two *Sargassum* species are pelagic (their entire life cycle occurs on the sea surface): S. fluitans (morphotype III) and S. natans (morphotypes I and VIII) (Butler et al., 1983). In the open ocean, *Sargassum* (hereby used to refer to pelagic *Sargassum* spp.) constitutes an essential habitat, refuge, food, or spawning for more than 120 species (Rodríguez-Martínez, Van Tussenbroek & Jordán-Dahlgren, 2016; Uribe-Martínez et al., 2020). The abundant presence of *Sargassum* in the Sargasso Sea has been known since the 15th century; however, in recent years, the presence of *Sargassum* has increased in the Caribbean sea due to a recurrent great Atlantic *Sargassum* belt (GASB) that has been observed in satellite imagery since 2011, often extending from West Africa to the Gulf of Mexico (Wang et al., 2019). Although *Sargassum* is important in the open ocean, several scientific studies confirm the severe damage of large concentrations of *Sargassum* caused to flora and fauna of coastal ecosystems (Rodríguez-Martínez et al., 2019), the health of the inhabitants of coastal towns (Resiere et al., 2019), and the economies of the cities and countries affected by the phenomenon (Marx, Roles & Hankamer, 2021).

Traditionally, open sea monitoring of *Sargassum* has been carried out using satellite remote sensing techniques. The first observation of *Sargassum* from space was recorded in 2005 (Gower et al., 2006). Due to large quantities of *Sargassum* reported in recent years in the central Atlantic Ocean and the Caribbean Sea and the frequent presence on the Caribbean beaches, studies on the monitoring of *Sargassum* in the open ocean have increased. Some of the most frequent studies include the use of satellite data from NASA’s AQUA, TERRA, and Landsat platforms, as well as the Sentinel-2 of the European Space Agency (ESA). (Wang & Hu, 2020). Additionally, many studies have been conducted to estimate the concentration of chlorophyll and other photopigments dissolved in the water and support the calibration of indices used in remote sensing (Lazcano-Hernández et al., 2019). Currently, the most widely accepted remote sensing methodologies used worldwide for detecting pelagic *Sargassum* in the open ocean are the Floating Algae Index (FAI) (Hu, 2009) and Alternative Floating Algae Index (AFAI) (Wang & Hu, 2016). However, due to the high heterogeneity of the coastal zone, intrinsic limitations of satellite platforms, and the observation conditions (*e.g.*, clouds and their shadows, atmospheric influences, and sun glint), these techniques are not suitable for efficiently monitoring *Sargassum* on the beach because false positives are frequently present (Wang & Hu, 2020). On the other hand, although MODIS images are available daily, their spatial resolution (from 250 to 1,000 m) is a major restriction for detecting *Sargassum* on the beach, while for images with higher spatial resolution such as those provided by Landsat 8 (from 15 to 30 m) their major restriction is their temporal resolution (16 days). Hence, we suggest that conventional remote sensing requires accurate ground information at the beach level.

Deep learning (DL) is a powerful emerging technology that has recently evolved in the field of Machine Learning (ML). It has proven to be significantly superior to traditional statistical or physics-based algorithms for the extraction of features and information from images in many industrial fields applications (Li et al., 2020) and is beginning to awaken interest in ocean remote sensing applications (Arellano-Verdejo, 2019; Wang & Hu, 2021). One of the first coastal monitoring approaches that used DL for automatic analysis of features of MODIS imagery was ERISNet (Arellano-Verdejo, Lazcano-Hernandez & Cabanillas-Terán, 2019). In this study, a deep neural network (ERISNet) was explicitly designed to detect *Sargassum* in Aqua-MODIS imagery, achieving 90% accuracy in their classification skills after training. However, given the spatial resolution of the MODIS images, accurate monitoring along the beach is not feasible. Another novel approach to coastal monitoring, and in particular to monitor *Sargassum* on the beach, is Collective View (Arellano-Verdejo & Lazcano-Hernández, 2021), which maps the presence/absence of *Sargassum* after automatic classification of images uploaded by citizens through the scheme of Crowdsourcing (Arellano-Verdejo & Lazcano-Hernandez, 2020). Although the proposal is innovative, the presence/absence maps provide limited information and do not facilitate decision-making.

To address the challenge posed by *Sargassum* on the beaches, it is essential to have sufficient information, such as the area of coverage or, ideally, the volume of biomass accumulated on the beach for creating efficient cleanup strategies. However, existing remote sensing methodologies cannot always be applied because of several technical or environmental factors. Several studies report manual measurements of *Sargassum*, the results of which were used to infer the amount of *Sargassum* in wider areas (Salter et al., 2020; García-Sánchez et al., 2020; Torres-Conde & Martínez-Daranas, 2020). However, due to the magnitude of the phenomenon, we believe that such techniques, although interesting, are hard to scale. We consider that a methodology that automatically quantifies the amount of *Sargassum* on the beach is still lacking. The quantitative estimation of *Sargassum* distribution along the coast can support the planning and quantification of the *Sargassum* collected and thus reduce the collateral effects it causes.

To contribute to the solution of the problem stated, the present study proposes *Sargassum* mapping along the beach using the percentage of *Sargassum* coverage in geotagged photographs to design the maps. Semantic segmentation (SS) was used to classify three elements within each picture (sand, *Sargassum*, and other elements). This provided a quantitative approximation of the *Sargassum* presence on the beach in each photograph. This methodology started from the premise that the accumulated *Sargassum* is found on the beach, so the study was carried out with pictures that met this condition. The automatic segmentation of *Sargassum* in the water requires work that is beyond the scope of this study. Finally, a choropleth map of *Sargassum* coverage area is proposed.

## Materials and Methods

The study area for the initial implementation of this methodology was Mahahual, Quintana Roo (18.715693, −87.708009), located in the southeast of the Yucatan Peninsula, in Mexico. We used geotagged photographs taken between September 14th, 2019 and August 24th, 2021. This area has received periodical massive landings of *Sargassum* since late 2014 with peak years occurring in 2015, 2018, 2019, and 2021 (Arellano-Verdejo & Lazcano-Hernández, 2021). The images collected to build the data set for this study were taken from the Collective View platform, which focuses on collecting photographs at the beach scale using the Crowdsourcing scheme (Arellano-Verdejo & Lazcano-Hernández, 2021). Technical, visual and temporal features of the photographs are beyond our control; however, even with these limitations, they proved to be a valuable source of input.

The following sections describe the semantic segmentation method, the Pix2Pix model, and the processes followed to build the segmented image dataset, which was then used to train the Pix2Pix neural network architecture for automatic image segmentation. The segmented images were used to generate maps showing the levels of *Sargassum* coverage captured by the photographs collected for the study region.

### Semantic segmentation

Semantic segmentation (SS) is an essential component in image processing and computer vision. SS has multiple applications, such as scene understanding (Hofmarcher et al., 2019), autonomous driving (Feng et al., 2020), and medical image analysis (Liu et al., 2020; Abedalla et al., 2021; Nguyen et al., 2021), among many others. The goal of SS is to change the representation of an image into something more meaningful and easier to analyze using post-processing algorithms. More specifically, SS can be formulated as a semantically labeled pixel classification problem. SS performs pixel-level labeling with a set of objects in specific categories (*e.g.*, people, cars, traffic lights, sidewalks) for all pixels in the image.

In recent studies, SS based on deep learning (DL) techniques have shown promising results. There are several approaches based on DL that have been used to perform SS. Essential approaches include Fully Convolutional Networks, Convolutional Models with Graphical Models, Encoder-Decoder Based Models, Multi-Scale and Pyramid Networks, R-CNN, Dilated Convolutional Models, Recurrent Neural Networks, Attention-Based Models, and Generative Adversarial Networks (Garcia-Garcia et al., 2017; Lateef & Ruichek, 2019).

In addition to the traditional approach, where SS consists of classifying each pixel of an image, SS can also be approached as the “translation” of an input image into a corresponding output image. This can be done using a Generative Adversarial Network designed for this specific purpose. Generative Adversarial Networks (GAN) are models that learn how to map from the input vector of random noise “z” to the output image “y” (Creswell et al., 2018). GANs are composed of two main blocks: the generator and the discriminator. In short, within GANs, the generator block learns to produce the desired output from a random noise input vector, and the discriminator block tries to understand if the incoming content has been created by the generator block (fake) or if it is real content; even further, conditional GANs(cGAN) generate their output from a random input vector, but unlike GANs, they also use a vector that conditions the output class (Gu et al., 2015; Gu et al., 2018).

The study (Isola et al., 2017) analyzed the use of cGAN as a general-purpose solution for image-to-image translation problems (Pix2Pix). While the network learns the mapping from the input image to the output image, Pix2Pix also enables it to learn the loss function necessary to perform this mapping. This is important as it avoids writing a loss function every time the problem changes. Consequently, the same approach can be applied to issues that initially require different loss functions, allowing for the application of this approach to segmentation problems.

The Pix2Pix architecture has proven effective in problems where the output is detailed or photographic concerning the input. Phillip Isola also applied this architecture using a small set of urban images to perform semantic segmentation where the output is less complex than the input. The results showed that Pix2Pix produced sharp photos that, at first glance, appeared to represent the terrain adequately but included some minor “artifacts” that made the results less than perfect (Isola et al., 2017).

One of the notable features of the Pix2Pix model is the quality of the output as a function of the size of the dataset used to perform the training. While classical SS models use large data datasets with thousands, even hundreds of thousands of images, Pix2Pix training image datasets are relatively small, including only 400 images for the facades example (Isola et al., 2017). Given the characteristics of the phenomenon and the information available for this study, we have used the Pix2Pix model as a mechanism for segmenting the images that show the presence of *Sargassum*.

### Segmented *Sargassum* dataset

SS algorithms must be trained and validated using large datasets. Like most real-world problems where access to datasets for training is not possible (or they do not exist), there is an absence of a dataset of segmented images of *Sargassum* on the beach in the literature and the principal repositories containing training datasets for SS algorithms.

**Figure 1:**
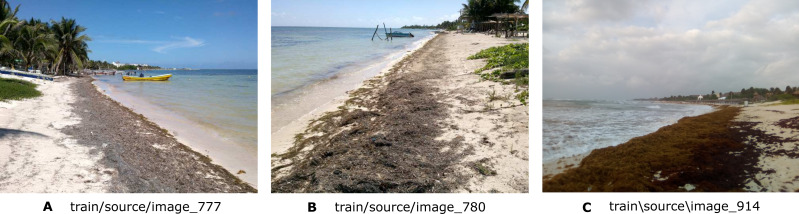
Three examples of images (A, B, and C) showing the presence of *Sargassum* on the beach. A1 to A3 show images that meet the desired framing and composition requirements. As it can be seen, the photos highlight the presence of *Sargassum* on the beach, the depth, and other elements, such as water, the sky and palm trees.

The above is probably because the unusual arrival of *Sargassum* on Caribbean beaches is an emerging problem, and there is not sufficient data yet. Therefore, one of this study’s initial and significant challenges was to create a segmented *Sargassum* dataset. The generation of this dataset was carried out in two stages: image collection and selection and image segmentation. The first stage consisted of selecting 1,000 images with the desired composition and framing the characteristics of the beach containing *Sargassum*. Concerning the composition of the photos, we selected those that contained elements that allowed for categorizations where some would gain more importance than others (Fig. 1).

In summary, a set of images was manually selected from Collective View (CV), in which *Sargassum* covered an important percentage of the photograph. CV is a crowdsourcing-based platform used to monitor the state of the beaches using images that show the presence of *Sargassum* on the beaches (Arellano-Verdejo & Lazcano-Hernández, 2021).

**Figure 2:**
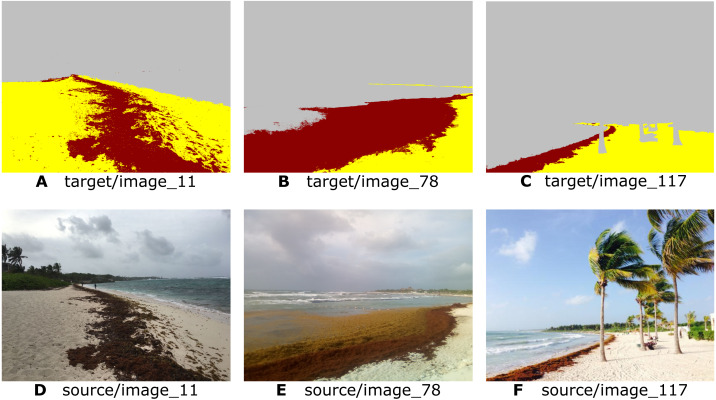
Examples of the segmented image dataset. The upper part (A to C) shows the segmented images, while the lower part (D to F) shows the original photos for each segmented image. As it can be seen, all of them have the desired composition and framing elements that were discussed previously.

The second stage consisted of manually segmenting the selected images to highlight three categories: *Sargassum*, Sand, and the other elements present in the image (Fig. 2). Due to the small size of the training set (1,000 images), this task was carefully performed by a person with experience in digital image editing to achieve the best possible quality in a reasonable amount of time. The time to build the data set was about six months.

Figure 2 shows three examples of the segmented image dataset created. After searching, selecting, and segmenting the images, a dataset containing 1,000 segmented *Sargassum* images was created. From the data set, 800 images were selected randomly for the training of the Pix2Pix algorithm, and the remaining 200 as a validation data set, used to assess the algorithm’s ability to segment images different from the ones it used previously (a generalization of the algorithm). This dataset is the first of its kind (https://doi.org/10.6084/m9.figshare.16550166.v1). It opens a range of opportunities to develop algorithms that help carry out multiple tasks, including estimating the presence of these macroalgae along the beaches.

Finally, given the dynamics of the observed phenomenon, in the images of the created dataset for this study, we cannot guarantee that there are the same amount of pixels for each of the classes that will be segmented, generating a natural imbalance in the dataset. The “F1 score” (described below), is used to assess the quality of the segmentation for each class and the weighted average for the overall performance of the algorithm. The weighting weights are based on the number of pixels present for each class in each photo (support).

### Pix2Pix for SS

Although the translation from one image to another is not formally a segmentation process, it is possible to obtain acceptable results in a reasonable time and with relatively small data sets for training, compared to classical segmentation algorithms. To calculate and estimate the percentage of *Sargassum* present on the beach in an image, we performed the translation of the image to be analyzed to the segmented image domain. Given that the resulting image does not strictly correspond to a segmented image, where it is ensured that each of the pixels corresponds to each of the classes (*Sargassum*, Sand, and the other elements), the translated image was processed using a k-means algorithm to assign each pixel a class (*k* = 3) and to obtain a segmented image finally.

k-means (Likas, Vlassis & Verbeek, 2003) is an unsupervised clustering algorithm whose objective is to group a data set *X* into *k* classes. Let *X* be a data set *X* = {*x*_1_, *x*_2_, …, *x*_*n*_}, *K* classes with *K* ≤ |*X*| and a set of clusters C ={c_1, c_2, …, c_k}, k-means is formally defined as a problem of minimization of variances within *C*-groups (squared Euclidean distances) as shown in the Eq. (1), where *μ*_*i*_ is equal to the mean of the elements *x*_*i*_ ∈ *c*_*i*_.

(1) \begin{eqnarray*}\text{arg min}_{\mathbf{S}}\sum _{i=1}^{k}\sum _{{\mathbf{x}}_{j}\in {c}_{i}}{ \left\| {\mathbf{x}}_{j}-{\mu }_{i} \right\| }^{2}.\end{eqnarray*}

Although there are several vector quantization techniques, mainly used in signal processing (Cosman et al., 1993; Nasrabadi & King, 1988; Buzo et al., 1980), k-means is a clustering technique that has been widely used in color segmentation in different problems and with good results (Zhang et al., 2014; Chen, Chen & Chien, 2008; Wu, Lin & Chang, 2007). We used the Pix2Pix model because it has shown promising results with small training data sets of a few hundred pictures and because it was not necessary to define a specific loss function for our problem since the Pix2Pix model could learn it automatically through the use of a GAN.

The Pix2Pix GAN architecture involved carefully specifying a generator model, a discriminator model, and a model optimization procedure. As frequently present in deep convolutional neural networks, both the generator and discriminator models used standard Batch Convolution-Normalization-layer blocks.

#### Generator

**Figure 3:**
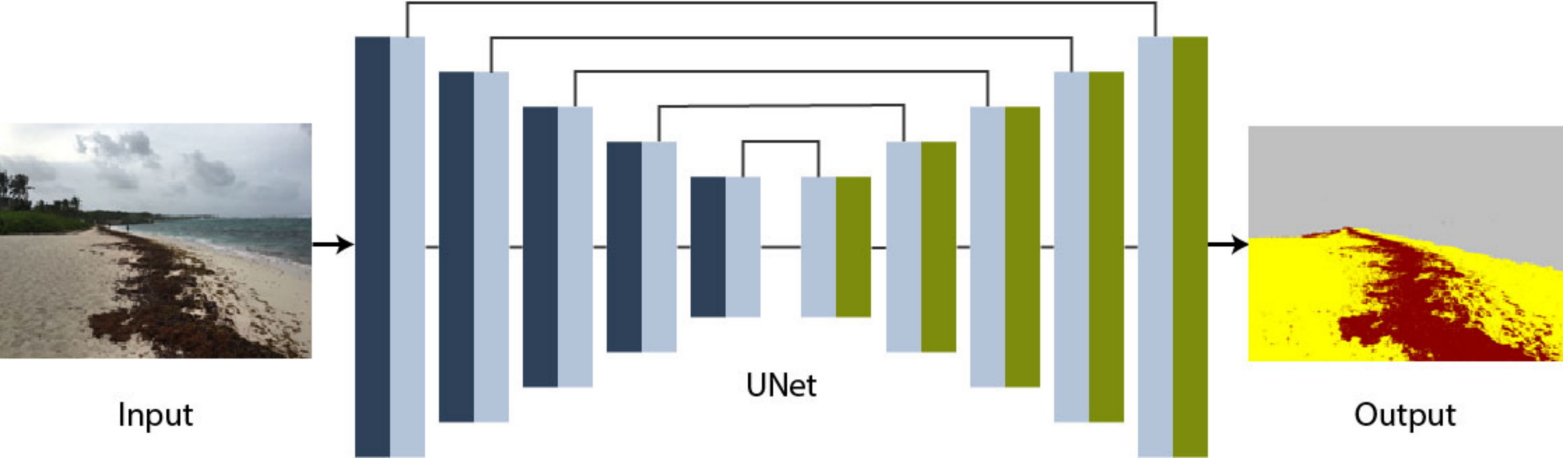
Pix2Pix generator based on UNet architecture. The RGB image is the input of the UNet and the output is the translated image.

In Pix2Pix, a generative model considers an image as input, and, unlike a traditional GAN model, it does not consider a point in latent space as input. Instead, the source of randomness comes from the dropout layers used both during training and when making a prediction. As described in the original paper (Isola et al., 2017), the generator we have used for the present study used a UNet type architecture (see Fig. 3).

The encoder–decoder generator architecture of a UNet-type architecture takes an image as input and reduces it through several layers until it reaches a bottleneck layer, where the representation is again increased in several layers before obtaining the final image with the desired size.

In the algorithm used in our study, the encoder used convolutional modules (C) with the following distribution: C64 - C128 - C256 - C512 - C512 - C512 - C512 - C512 - C512 with a LeakyReLU activation function (The number after the letter C indicates the number of filters used in the convolutional layer). The first block (C64) used a batch normalization function. Regarding the decoder, it used convolutional modules with the following distribution: CD512 - CD512 - CD512 - CD512 - C512 - C256 - C128 - C64 with a ReLu activation function. The first three blocks (CD512) used a Dropout function with a probability of 0.5. As suggested in Isola et al. (2017), all networks were trained from scratch. Layer weights were initialized from a Gaussian distribution with a mean of 0 and a standard deviation of 0.02.

To carry out the training process, the estimation of the generator loss function was computed as the average absolute error between the UNet output and the target image (*i.e.,* the difference between the generated image and the hand-segmented image found within the dataset that corresponds to the input image).

#### Discriminator

The discriminator model takes an image from the source domain and an image from the target domain and predicts the probability of the segments in the target domain image being a real or generated version of the source image. The discriminator model input highlights the need for an image dataset composed of matched source and target images when training the model. In this study, the set of images was composed of the original image containing *Sargassum* and the corresponding segmented image.

Unlike the traditional GAN model that used a deep convolutional neural network to classify images, the discriminator used by Pix2Pix is a Markovian type and was implemented using a PatchGAN type network. Unlike standard models, instead of returning a single value classifying the entire output (image) as real or fake, it produces a set of values where different original image patches are evaluated. This generates a grid where, for each grid cell, it considers whether the image patch is real or fake.

**Figure 4:**
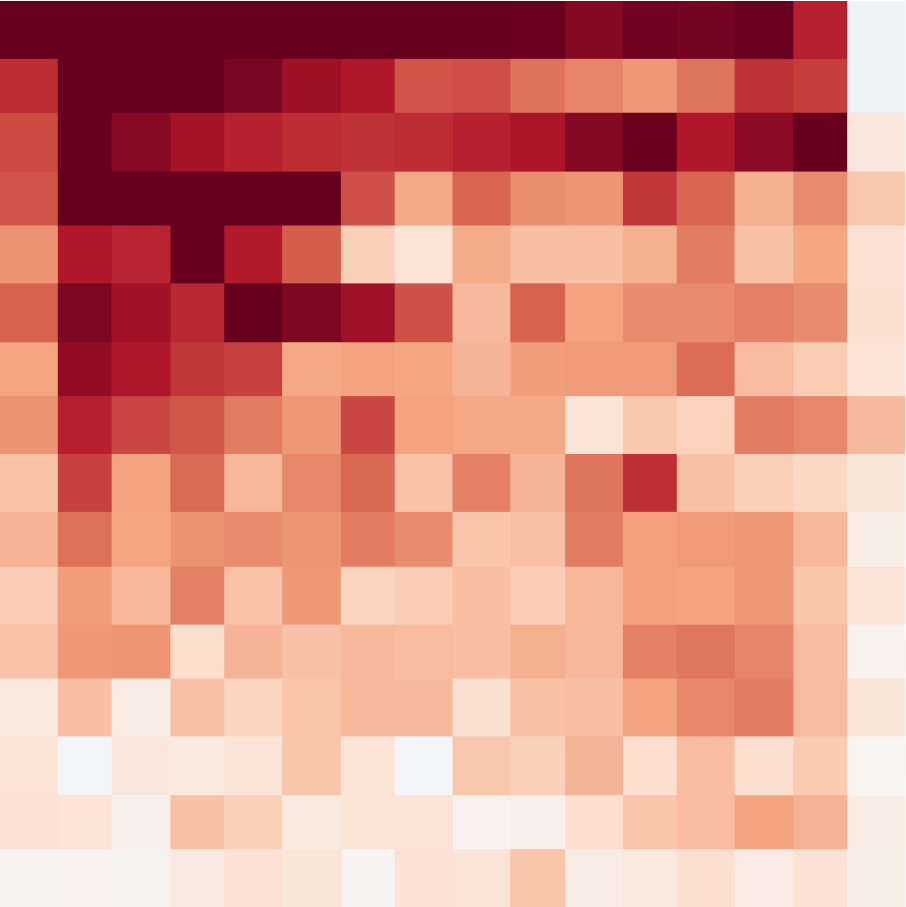
Pix2Pix matrix discriminator output (feature map of true/false predictions obtained at the output of the network).

The PatchGAN discriminator model can be implemented as a deep convolutional neural network. The number of layers is configured so that the effective receptive field of each network output can be mapped to a specific size in the input image. The model used in the present study uses a convolutional (C) architecture with the following distribution: C64 - C128 - C256 - C512 with a LeakyReLU activation function. As in the case of the generator, the layer weights were initialized from a Gaussian distribution with a mean of 0 and a standard deviation of 0.02. The network’s output is a single feature map of true/false predictions that can be averaged to obtain a single score (See Fig. 4).

To carry out the training process, the loss value of the discriminator was calculated as the sum of the differences between the real value and the value detected by the discriminator, in addition to the differences between the generated false values detected by the discriminator.

To train the Pix2Pix model, we used the Python3 programming language and Tensorflow 2.6 library (https://www.tensorflow.org/). The experiments were carried out in the cloud using Google Colab (https://colab.sandbox.google.com) as well as an execution environment using GPUs as processing units.

On the other hand, existing maps showing the presence of *Sargassum* on the beach only contain points that indicate the presence or absence of this macroalgae. Although these maps show valuable information, they are incomplete. More effective maps can be constructed to show *Sargassum* concentrations and thus can be used by decision-makers.

### *Sargassum* coverage maps

Maps are a fundamental tool for the study and visualization of phenomena with some spatial component; that is why they are applied in various areas of knowledge (Baniata, Mahmood & Kertesz, 2021; Ma, Zhang & Huang, 2021). Concerning the study of *Sargassum*, there are studies (Arellano-Verdejo & Lazcano-Hernandez, 2020; Arellano-Verdejo & Lazcano-Hernández, 2021), that present an approach to the generation of *Sargassum* maps on the beach. In their work, the authors offer a methodology for acquiring images based on Crowdsourcing, processing these images, and finally, creating maps that show the presence/absence of *Sargassum*. However, despite the innovation of this proposal, it does not thoroughly help decision-making.

This study aims to take *Sargassum* mapping to the next level by developing coverage maps of *Sargassum*, fed with the values of the *Sargassum* extent area calculated for each of the geotagged photographs of the beach. With the estimation of *Sargassum* coverage in the photographs, we consider that it is possible to improve available maps (Arellano-Verdejo & Lazcano-Hernández, 2021).

Based on the features of the study, to prepare the coverage maps, the following analyses were carried out: (I) To find out the type of distribution of the data used in the study, the Kolmogorov–Smirnov test was used to evaluate the normality of the data. (II) To perform a comparative analysis with open data from Landsat satellite platforms, the tessellation was built with contiguous square polygons at 30 m, which coincide with the location and pixel size of the sensors of the Landsat platform (OLI / TIRS). (III) The pixel’s color is related to the percentage of *Sargassum* of the photo within the pixel. (IV) In the case of two or more photographs per pixel, the mean of the coverage values of the pixels in the photographs will be taken.

As far as we know, there are no *Sargassum* coverage maps on the beach that use quantitative methods for their design. There have only been qualitative approximations that lack statistical confidence. Thus, one of the main contributions of this study is to generate *Sargassum* coverage maps that show a first approximation of the percentage of *Sargassum* in the observation area through a quantitative and replicable method.

### Exploratory data analysis

For the exploratory analysis of the results of the metrics used to determine the performance of the proposed algorithm, a box plot was used, which is a standardized method to represent numerical data graphically through quartiles. In this way, the median, the distribution of the data in quartiles, and their outliers are shown at a glance. A box plot includes the following elements: range, outliers, quartiles (Q1, Q2, and Q3), median (Q2), as well as minimum and maximum values. The lines extending from the box to the minimum and maximum values are called whiskers. The values between the lower whisker and the lower limit of the box represent 25% of the total values and are less than the minimum value of the standard deviation, which is represented by the lower limit of the box and is also known as quartile 1 (Q1). The values within the box are those that fall within the range of the standard deviation and represent 50 percent of the total values. The line inside the box corresponds to the median and is also known as quartile 2 (Q2). The values between the upper limit of the box and the upper whisker correspond to 25% of the values and are greater than the upper limit of the standard deviation, which is represented by the upper limit of the box, also known as quartile 3 (Q3). Finally, the values out of the whisker limits belong to outliers.

### Metrics used for the assessment of the model

**Figure 5:**
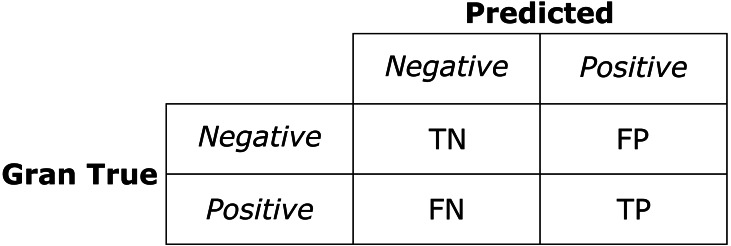
Basic structure of 2 * 2 confusion matrix.

The confusion matrix (Fig. 5) is a tool used to visualize the performance of a “supervised learning” algorithm. Each column of the matrix depicts the number of predictions in each class, while each row represents the instances in the actual class. The confusion matrix allows us to visualize the hits and misses of the model, condensing the results into four categories: True Positive (TP), True Negative (TN), False Positive (FP), and False Negative (FN).

Within Machine Learning, several metrics allow for evaluating the relationship between the values classified correctly and those classified incorrectly by a given model (Hossin & Sulaiman, 2015). In this study, we used the Accuracy, Precision, Recall, and F1 Score metrics. Accuracy evaluates the ratio between the correctly classified values and the total number of cases (Eq. (2)).

(2) \begin{eqnarray*}\text{Accuracy}= \frac{TN+TP}{TN+FP+FN+TP} .\end{eqnarray*}

The Precision metric serves to evaluate the performance of a model when false positives are relevant for the study. This metric is computed as the quotient between True Positives and total predictions, as shown in Eq. (3).

(3) \begin{eqnarray*}\text{Precision}= \frac{TP}{TP+FP} .\end{eqnarray*}

On the other hand, the Recall metric serves when false negatives acquire a relevant role within the context of the study, evaluating the model performance based on the FN obtained. The Recall is calculated as the quotient of the True Positives between the sum of True Positives and False Negatives (Eq. (4)).

(4) \begin{eqnarray*}\text{Recall}= \frac{TP}{TP+FN} .\end{eqnarray*}

The F1 Score (Eq. (5)) is used when it is required to maintain a trade-off between Accuracy and Recall; for example, when both FPs and FNs have equal relevance in the context of the study. Eq. (4) shows the F1 Score.

(5) \begin{eqnarray*}F1\text{Score}= \frac{\text{Precision}\ast \text{Recall}}{\text{Precision}+\text{Recall}} .\end{eqnarray*}

The value computed for all the above metrics is a Real number in the interval [0,1], where 1 represents an ideal model and 0 is the worst case. These values are also usually expressed as a percentage.

Finally, a technique commonly used to evaluate object detection and SS algorithms is called Intersection over Union (Rahman & Wang, 2016). The Jaccard similarity coefficient (Eq. (6)), also known as IoU or intersection over the union, can be an alternative to the aforementioned metrics, like an F1 Score. The IoU is a statistic used to measure the similarity and diversity between A and B sets, where A is the set of pixels of the manually segmented image, and B is the set of pixels of the output obtained by the methodology proposed in this study.

(6) \begin{eqnarray*}J(A,B)= \frac{{|}A\cap B{|}}{{|}A\cup B{|}} = \frac{{|}A\cap B{|}}{{|}A{|}+{|}B{|}-{|}A\cap B{|}} .\end{eqnarray*}

## Results and Discussion

The Accuracy metric of the classification algorithm (Eq. (2)) tends to overestimate the result. Because of this, we used other accuracy metrics such as: Precision, Recall, and F1-Score (Hossin & Sulaiman, 2015). We computed the Precision, Recall, and F1-Score metrics over the testing dataset (20% of the whole dataset).^1^ 1Test folder of the dataset: https://figshare.com/articles/dataset/Sargassum_Segmented_Dataset/16550166/1.Table 1 shows the mean, standard deviation, maximum, minimum, and quartile values.

**Table 1:** Measurements on the validation dataset (200 images).

	**Precision**	**Recall**	**F1 Score**
**mean**	**0.93**	0.91	0.91
**Std. Dev**	0.04	0.07	**0.06**
**min**	0.74	**0.44**	0.48
**25%**	0.91	0.89	0.89
**50%**	0.94	0.93	0.93
**75%**	0.96	0.95	0.96
**max**	**0.99**	0.99	0.99

**Figure 6:**
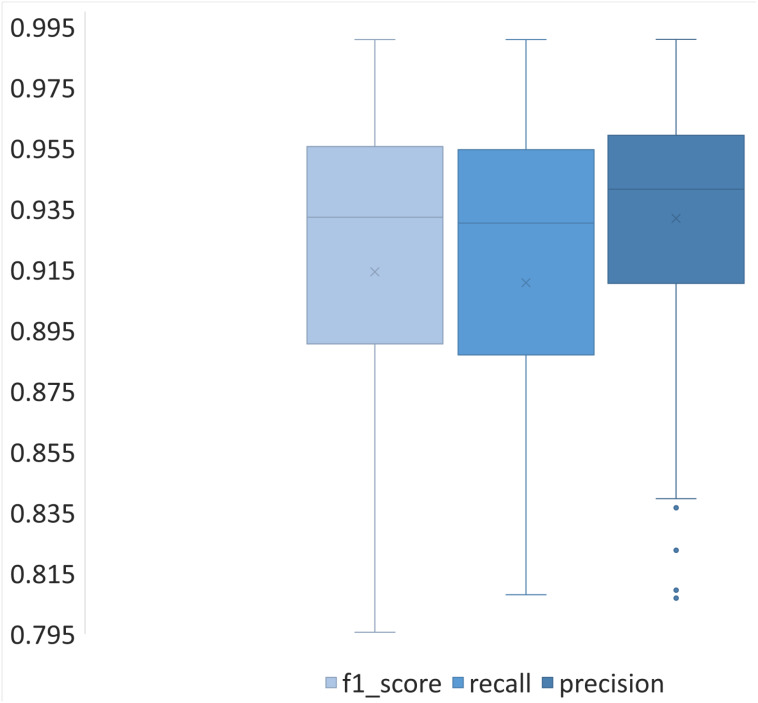
Box plot for F1 Score, Recall, and Precision metrics, the values derived from applying each metric to the validation dataset (200 images).

In Fig. 6, we can see that the Precision metric obtained the highest value median and the lowest dispersion (smallest standard deviation), followed by the F1 score, and finally, the Recall metric. We can also observe that in all the cases, the lower whiskers are longer than the upper whiskers, indicating that there is a negative bias in the values. Despite the small differences between the metrics, the evaluation of the proposed algorithm is similar.

Due to the diversity of the analyzed images, there were not a similar number of pixels for each category (*Sargassum*, Sand, and others); thus, we did not have a balanced dataset. This diversity may be positive since it induced stress during the training process, favoring the generalization of the final segmentation model. This could help understand why the metrics’ average performance is similar. The following section presents the metrics results: Precision, Recall, and F1 score, calculated for the segmented images corresponding to the best, average, and worst cases.

### Model accuracy assessment

Precision is the ratio of the number of true positives to the total number of true and false positives; for instance, the total number of values predicted as positive. Precision is a measure to determine when false positive play an important role in the context of the classification problem, which would be interesting depending on the category predicted (*Sargassum*, Sand, and others). On the other hand, Recall shows the relationship between true positives and the sum of true positives plus false negatives; thus, it computes how many true positives our model captured by labeling it as positive (true positive). This metric is useful when the cost associated with the false negative is high. Finally, the F1 value is used to combine the accuracy and recall measures into a single value. This is practical because it makes it easier to compare the combined performance of accuracy and completeness between various solutions.

**Figure 7:**
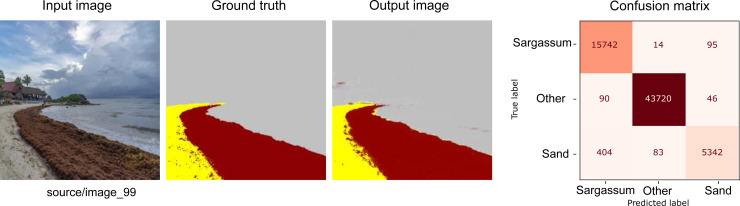
From left to right: the Input image (RGB), the Ground truth (manually segmented), the Output (segmented by the proposed methodology), and the confusion matrix, which allows the visualization of the algorithm performance regarding the Input image, which in this case corresponds to the best-evaluated image.

**Figure 8:**
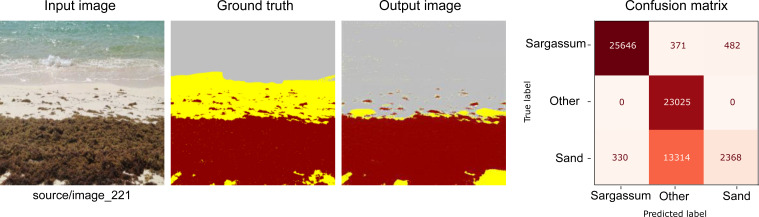
From left to right: the Input image (RGB), the Ground truth (manually segmented), the Output (segmented by the proposed methodology), and the confusion matrix, which allows the visualization of the algorithm performance regarding the Input image, which in this case corresponds to the worst evaluated image.

**Figure 9:**
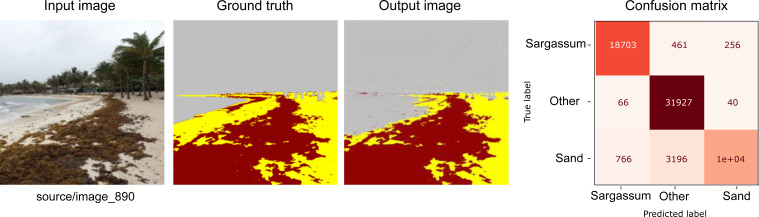
From left to right: the Input image (RGB), the ground truth (manually segmented), the Output (segmented by the proposed methodology), and the confusion matrix, which allows visualizing the performance of the algorithm with respect to the Input image, which in this case corresponds to the image with an average evaluation.

Figures 7, 8 and 9 show three groups with three images in each one. The image on the far left corresponds to an RGB input from the CV platform. From left to right, the second image corresponds to the result of the segmentation carried out manually. Further, on the right, the image automatically segmented by our proposal is presented. Finally, the image at the extreme right corresponds to the confusion matrix. Additionally, the section colored in yellow corresponds to the sand on the beach, the area in brown corresponds to *Sargassum*, and the rest of the elements are in gray.

Figure 7 shows the result of the segmentation process for one of the ideal cases where the *Sargassum* is accumulated along the beach in a uniform and precise manner. In this first group of images, we can observe that the *Sargassum* was adequately segmented; however, the network had some inaccuracies with the vegetation in the background. Also, these images demonstrate how the categories are not balanced, which is reflected in the support column in Table 2. The number of pixels corresponding to the “other” category is higher than the rest. This has important implications when interpreting the confusion matrix and the results obtained by the metrics shown in Table 2.

As shown in the confusion matrix in Fig. 7, both the percentages of false positives and false negatives are lower when compared to those classified correctly, which is reflected in the metrics shown in Table 2. The weighted average for Precision, Recall, and the F1-Score is 99%; this weighting aimed to reduce the impact of the difference in support.

Figure 8 shows one of the worst cases. As one can see, the input image has a closed frame that does not provide information regarding the accumulated *Sargassum* on the beach. Because of the features of this image, the algorithm cannot be used to identify the difference between the Sand and water pixels. Due to mentioned above, these kinds of images were not used to elaborate *Sargassum* coverage maps.

As shown in Table 3, the low values for the F1 score metric are only present for the “Sand” and “other” classes. However, this value is not due to the imbalance of the number of pixels; it is due to the number of False Positives and False Negatives produced by the algorithm for this image. As observed in the output image, most of the pixels of the Sand category were confused with the “other” category. That generates a negative effect reflected in the Recall value for the Sand category; for instance, these values classified as false positives directly impact this metric.

**Table 2:** Metrics for the best result (Fig. 7).

	**Precision**	**Recall**	**F1 Score**	**Support**
***Sargassum***	0.99	0.99	0.98	15851
**Other**	1.00	1.00	1.0	43856
**Sand**	0.97	0.92	0.94	5829
**accuracy**			0.99	65536
**macro avg**	0.98	0.97	0.97	
**weighted avg**	0.99	0.99	0.99	

**Table 3:** Metrics for the worst result (Fig. 8).

	**Precision**	**Recall**	**F1 Score**	**Support**
***Sargassum***	0.99	0.97	0.98	26499
**Other**	0.63	1.00	0.77	23025
**Sand**	0.83	0.15	0.25	16012
**accuracy**			0.78	65536
**macro avg**	0.82	0.71	0.67	
**weighted avg**	0.82	0.78	0.73	

It is essential to mention that the algorithm detected the *Sargassum* pixels with an accuracy of 96%; nevertheless, given that the calculation of the coverage area required a better classification, not only of the *Sargassum* but also of the Sand in general, the result of this image was considered unsatisfactory.

As shown in Table 4, the “macro avg” and “weighted avg” values for the “F1 score” were 91% and 92%. Figure 9 shows the result for an image that is within this average. Unlike Figs. 8 and 9 (as in Fig. 7) offers a beach perspective that contributes to inferring the state of the accumulated *Sargassum* on the beach. In addition, these types of images help reduce the number of False Positives and False Negatives in the algorithm.

**Table 4:** Metrics for the mean result (Fig. 9).

	**Precision**	**Recall**	**F1 Score**	**Support**
***Sargassum***	0.96	0.96	0.96	19420
**Other**	0.90	1.00	0.94	32033
**Sand**	0.97	0.72	0.83	14083
**accuracy**			0.93	65536
**macro avg**	0.94	0.89	0.91	
**weighted avg**	0.93	0.93	0.92	

As shown in Table 4, the “macro avg” and “weighted avg” values for the“F1 score” were 91% and 92%. Figure 9 shows the result for an image that is within this average. Unlike Figs. 8 and 9, (as in Fig. 7) offers a beach perspective that contributes to inferring the state of the accumulated *Sargassum* on the beach. In addition, these types of images help reduce the number of False Positives and False Negatives in the algorithm.

As demonstrated in Table 4, and like in the previous cases, the categories were not balanced. We can also see again that the algorithm had some issues incorrectly classifying the pixels of the Sand category and confused them with the water pixels. Because the water clarity makes it possible to see the seabed, the algorithm requires a higher contrast between classes to distinguish them.

Using the Precision, Recall, and F1-Score metrics, one can observe that, in general, the algorithm performed well in classifying the pixels corresponding to the *Sargassum* category (Valentini & Balouin, 2020). We can also see that under certain lighting circumstances and given the optical features of the water, in some cases, the algorithm tends to confuse the pixels related to the Sand. One of the most noteworthy results was the diversity of the images input and consequently the imbalance of the resulting categories. Given the above, the metrics used should be interpreted with discretion.

As observed in Table 5, the average value of the IoU metric is lower than the average value obtained by the F1-Score, decreasing from 91% to 85%. Nevertheless, the standard deviation is higher, implying a greater dispersion of the results. Finally, we can see that both values are similar in the case of the maximum value. In conclusion, the results obtained by IoU continue to be encouraging.

**Table 5:** Comparative assessment of the F1 and IoU metrics regarding the performance of the proposed algorithm.

	**F1 Score**	**IoU**
**mean**	0.91	0.85
**std**	**0.06**	0.09
**min**	0.48	**0.31**
**25%**	0.89	0.81
**50%**	0.93	0.88
**75%**	0.96	0.92
**max**	**0.99**	0.98

### Outliers

According to the basic analysis of dispersion and central tendency of the 200 images used in the validation stage, twelve images were identified as outliers by at least one of the metrics used in the algorithm performance analysis. The images that constitute the group of outliers contained one or more of the following features: few or no elements that allow the depth of the photographed landscape to be observed, rocks mixed with *Sargassum*, shadows, and shallow oligotrophic waters. Also, the fact that there was an unbalanced data set, with few Sand elements and a wide diversity of surfaces that were categorized as “others” gave rise to the following categories in the classification: (i) pixels corresponding to others were classified as Sand or *Sargassum* and (ii) pixels corresponding to Sand were classified as others. *Sargassum* was classified as *Sargassum* with a high level of confidence since it was a constant and predominant element in the images, so the algorithm had sufficient information to classify it.

**Figure 10:**
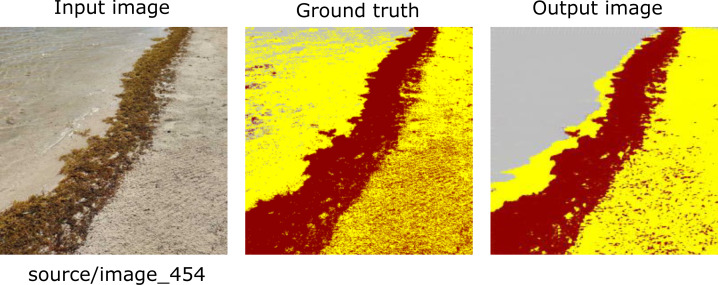
Example of Outlier, image_454. In the upper left corner of the input image can be seen transparent water, which allows to see the sand at the bottom; in the output image it can be observed that the algorithm classifies it in the “other” class. In the lower right corner of the Output image, it can be observed that the algorithm classifies better the marks of the rakes used for cleaning the beach compared to the Ground truth image.

Additionally, the outliers allow us to appreciate the performance of the algorithm from another perspective; for example, Fig. 10 shows image 454 (Input image),^2^ 2Dataset test folder: https://doi.org/10.6084/m9.figshare.16550166.v1.the image segmented manually (Ground truth), and the one segmented by the algorithm (Output image). In this case, the output image shows several pixels classified as ”others”, while the manually classified image indicates the presence of Sand. This is a debatable situation since the shallow depth and transparency of the water shown in the image allows us to see the Sand in the background. However, it is also true that what is on the Sand is water, so classifying it as “others” is also a correct. Although a lower number of pixels classified as “Sand” in the image increases the final percentage of *Sargassum* coverage on the Sand for this image it does not affect this study either statistically or substantially.

**Figure 11:**
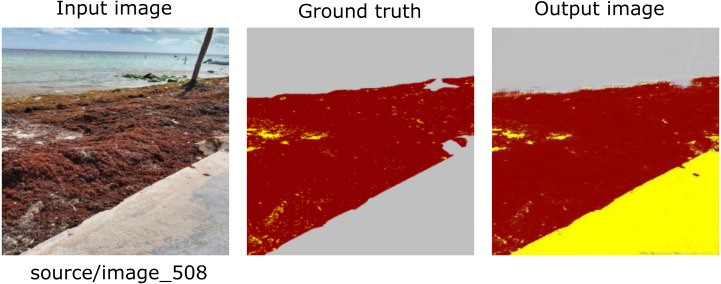
Example of Outlier, image 508. In the lower right corner of the output image, you can see that the algorithm classifies part of the concrete sidewalk as sand, since there is scattered sand in that zone. The outliers were poorly evaluated by the metrics because of the differences concerning the reference image (Ground truth). However, as can be seen, this does not necessarily mean that the image has been poorly segmented.

In the case of image Fig. 11, the output image shows several pixels classified as ”Sand”, while the manually classified image indicates the presence of ”other”. Again, this situation can have two valid points of view because there is Sand scattered on the sidewalk. Thus, classifying it as “Sand” is also suitable. More significant numbers of pixels classified as “Sand” in the image decrease the final percentage of *Sargassum* coverage. However, it is not critical and does not substantially affect this study. It allows us to observe the areas of opportunity that will allow the improvement of this algorithm. This proposal complements the information provided by the presence/absence maps (Arellano-Verdejo & Lazcano-Hernández, 2021) adding a quantitative value for estimating *Sargassum* cover.

### *Sargassum* distribution coverage maps

For calculating the *Sargassum* coverage on the beach in the photographs, the following procedure was implemented: (1) the geotagged images with the presence of *Sargassum* were segmented by the Pix2Pix network; at the end of this stage, the images were transformed, obtaining subtle differences in the color shades in pixels of similar classes. (2) These images were subsequently processed through the k-means algorithm, grouping the pixels into three categories: Sand, *Sargassum*, and “others” (3) From the segmented images, the pixels of the “others” category were discarded, and the sum of the pixels of the Sand and *Sargassum* categories were considered 100%. From this universe, the percentage of *Sargassum* on the beach was estimated in every photograph. (4) A shapefile composed of a tessellation of 30 × 30 m square polygons. Each of these tessellation polygons contains the *Sargassum* coverage value calculated by the segmentation process of the images associated with that polygon. (5) Finally, the *Sargassum* distribution map was built.

**Figure 12:**
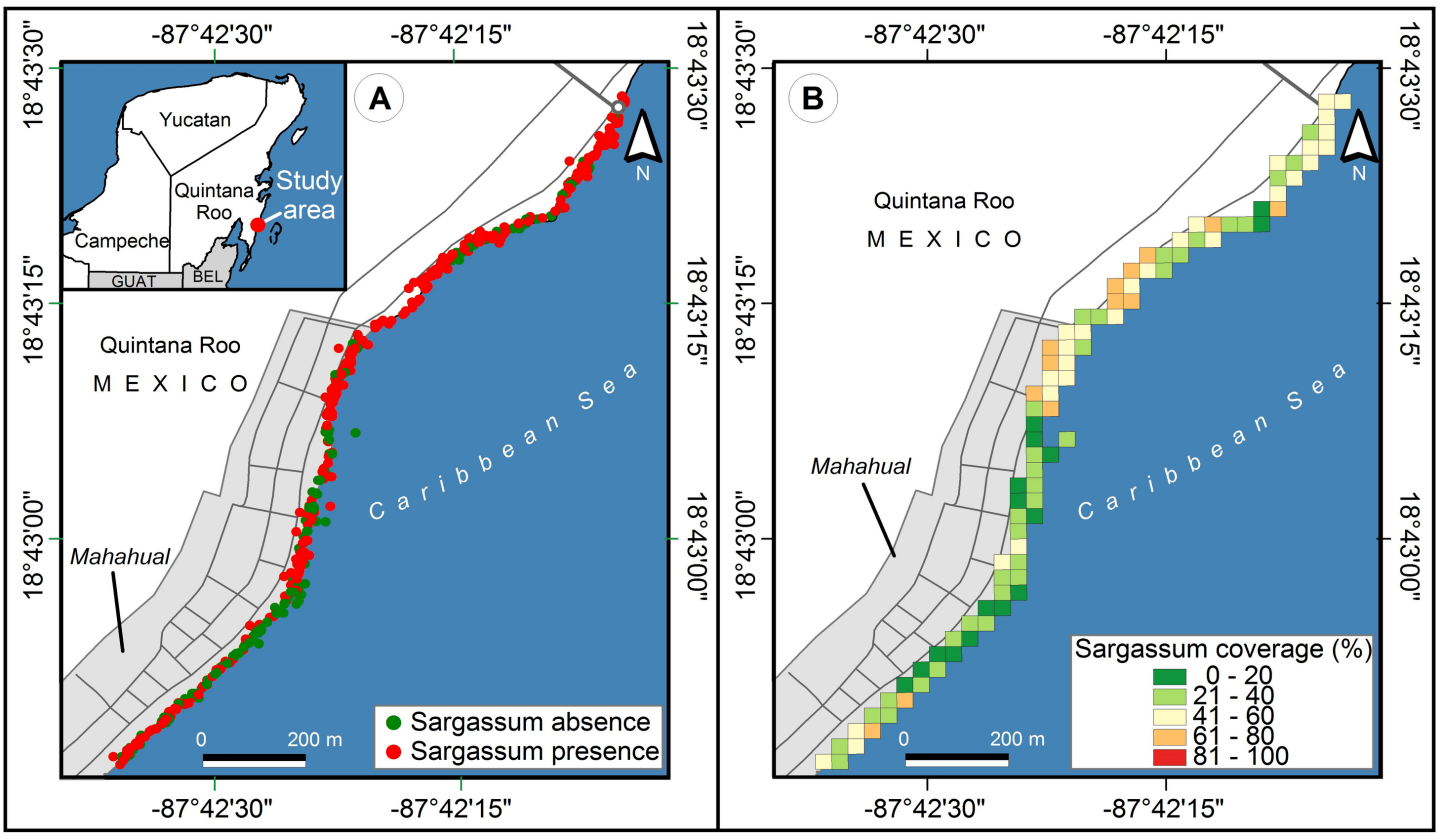
Study area, Mahahual, Quintana Roo (18.715693, −87.708009), located southeast of the Yucatan Peninsula, in Mexico. (A) The points represent the place where every picture was taken, green points indicate *Sargassum* absence and red points indicate *Sargassum* presence. (B) *Sargassum* coverage is shown using five colors, each representing a different percentage. Shades of green represent less presence of *Sargassum* than shades of red. (Source credit to Holger Weissenberger).

Polygons of 30 × 30 meters were selected as tessellation (according to the pixels of the Landsat 8 platform) for subsequent analysis and comparisons since it is one of the most widely used open data satellite platforms. The color of the polygons was defined by five levels of the color scale according to those traditionally used in most traffic lights around the world: green, yellow, and red; and two intermediate values, between green and yellow, and between yellow and red (see Fig. 12B). The colors with green tonalities correspond to zones with low levels of *Sargassum* accumulation. The yellow tonality is related to zones with moderate amounts of *Sargassum* presence. Finally, the zones with red tonalities correspond to zones with large areas of accumulated *Sargassum*. The average coverage area of the images within each polygon was used to set the color of each pixel.

Figure 12A shows an implementation of the *Sargassum* presence/absence map proposed by the “Collective View” study (Arellano-Verdejo & Lazcano-Hernández, 2021), where 642 photographs—different from those used in the Pix2Pix training—were selected from the Collective View platform at various points along Mahahual beach, Q. Roo, on 13 different dates between September 14th, 2019, and August 24th, 2021. Although this first proposal for *Sargassum* presence/absence mapping is innovative and represents an essential step in the quantitative mapping of *Sargassum* on the beach, its usefulness is minimal since it is infeasible to carry out a precise strategy with presence/absence data. More information is required to identify whether the presence of *Sargassum* refers to a small quantity, which does not represent an issue, or to an excessive amount, that represents a real challenge. Hence, this type of map does not allow for identifying the most affected areas.

**Figure 13:**
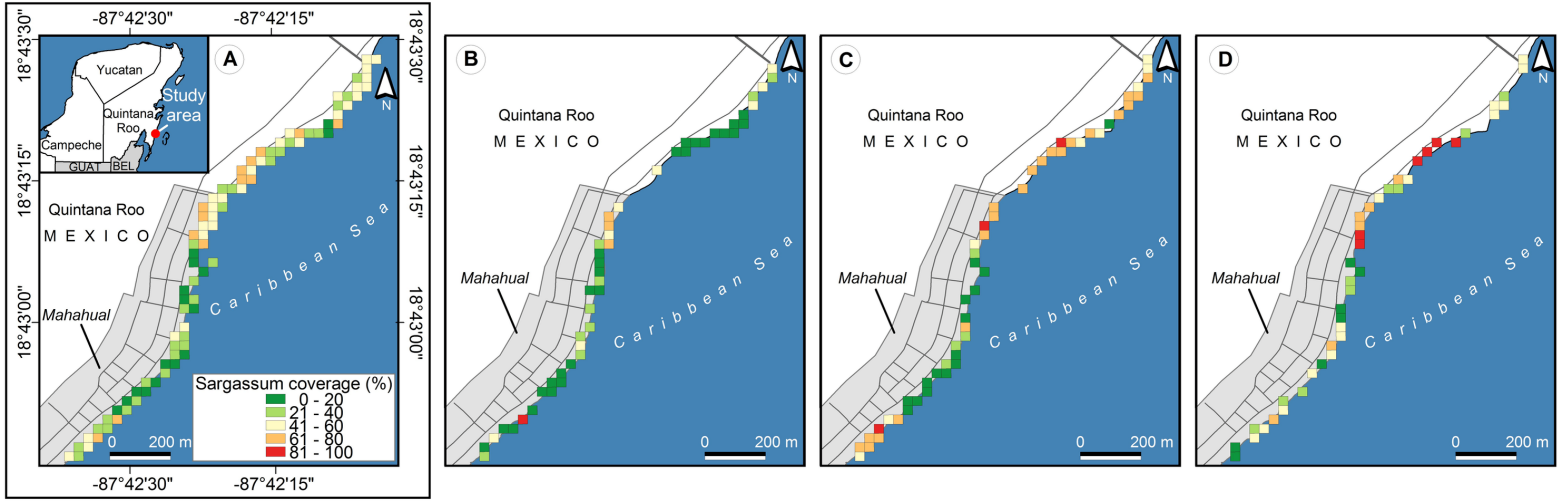
(*Sargassum*) cover maps on the beach of Mahahual, obtained through calculation of (*Sargassum*) cover percentage within 642 geotagged photographs, which were taken on twelve different dates between September 14, 2019, to August 24, 2021. (A) 12 full dates, (B) Photographs corresponding to December 16, 2020, (C) April 21, 2021, (D) August 24, 2021. (Source credit to Holger Weissenberger).

On the other hand, Fig. 12B shows the proposed map of this study, in this case, a tessellation built with contiguous square polygons at 30 m per side. A periodic and systematized sampling of the study area is required to carry out an accurate spatiotemporal study. However, the information extracted from the 642 photographs by semantic segmentation is more significant than that offered by Arellano-Verdejo & Lazcano-Hernández (2021) (at the time of writing this manuscript). According to the *Sargassum* coverage scale in Fig. 12B, we can observe that during the 23 months, the distribution of *Sargassum* was diverse along the study area and, as discussed in the analysis of Fig. 13, it coincides with the inter-annual variations described in other studies at larger scales (Wang et al., 2019).

In Fig. 12B, there are smaller amounts of *Sargassum* on the beach in front of the city of Mahahual and higher amounts to the north of the city. Periodic visits to the site during periods of *Sargassum* upwelling, as well as continuous communication with the people of the community of Mahahual, have given us the knowledge to surmise that the following factors can support us to interpret what the historical map shows.The first is that merchants and service providers clean their beaches periodically along the beaches located in the town. The second factor is a crack in the Coral reef in front of the beach in the northern area of the town, which causes a free passage of the *Sargassum* to the beach precisely in that area. Furthermore, the geomorphology of the coastline also plays an important role in determining the accumulation of greater amounts in the north of the city. Previous studies have already reported that beaches in bays and inlets tend to accumulate more *sargassum* than headlands (Salter et al., 2020).

From 2011 to date, variations have been observed in the time and the amount of *Sargassum* that arrives each year on the Caribbean coast. Significant amounts of *Sargassum* is observed each year in spring and summer and lower amounts in autumn and winter (Wang et al., 2019). These temporal patterns coincide with what is observed in the maps presented in Fig. 13. Figure 13A shows the average percentage of *Sargassum* coverage between September 14, 2019, and August 24, 2021. Maps Figs. 13B, 13C, and 13D show the average coverage of *Sargassum* for the dates corresponding to December 16, 2020, April 21, 2021, and August 24, 2021, respectively. With the support of the color scale, we can see that the coverage of *Sargassum* is lower in map Fig. 13B, which belongs to the autumn-winter season. On the other hand, Maps Figs. 13C and 13D belong to the spring-summer season (interannual increase in the presence of *Sargassum*), and that they contain more places with higher coverage values than map Fig. 13B.

As observed in the maps shown, the estimation of the area of *Sargassum* coverage within the images obtained through the Collective View platform, under the Citizen Science scheme, marks a breakthrough in the mapping of accumulated *Sargassum* coverage on the beach. As mentioned throughout this study, the proposed methodology complements the existing tools previously used for monitoring *Sargassum* on beaches. One aspect to consider regarding the proposed methodology is that the SS of different species of algae and seagrasses is a complex task as other seaweeds and seagrasses may be found on beaches with stranded *Sargassum*. Therefore, the proposed methodology may overestimate the amounts of *Sargassum* segmented in the images mainly at the beginning and end of the season. Therefore, to minimize this possible overestimation, the coverage values used in the design of the maps are grouped into five categories, each divided into equal intervals of 20%. Hence, if an overestimation occurs, the probability that it will show up in the maps is minimal.

**Figure 14:**
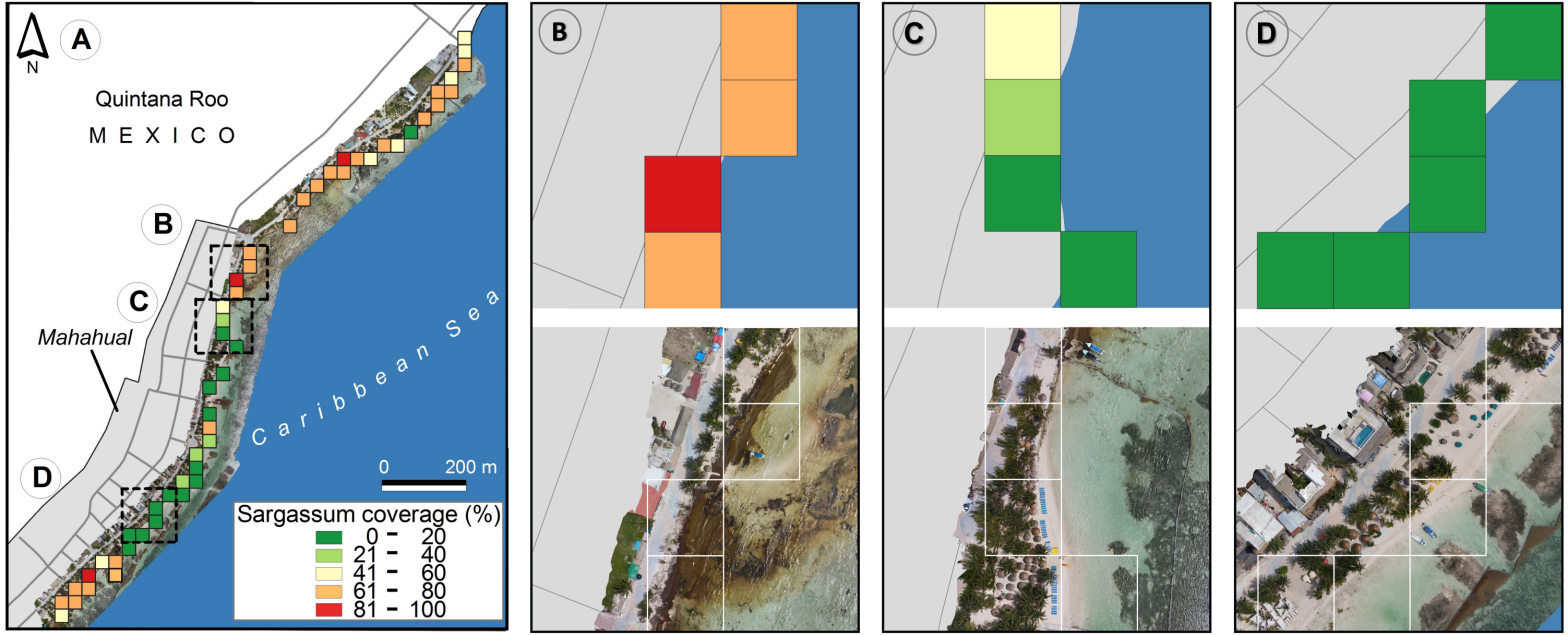
The study area of Mahahual beaches. (A) The proposed map and the orthophoto corresponding to April 21, 2021., (B) Close-up of the B zone, (C) Close-up of the C zone, and (D) Close-up of the D zone. (Source credit to Holger Weissenberger).

Figure 14A shows the proposed map for the study area, the orthophoto, and three zones (B, C, and D) approached to facilitate photo interpretation. The orthophoto used to elaborate Fig. 13 was generated with the Open Drone Map (ODM) software (https://www.opendronemap.org/webodm/). In all, 422 photographs were taken with a “DJI Air 2” drone. “Dronelink” (https://www.dronelink.com/) application supported autonomous flight. The main parameters for the mapping were the following: flight altitude of 56.2 m, vertical displacement speed of 16 km/h, front overlap 80%, side overlap 70%, picture shot rate 2 s; from these parameters, the obtained GSD was two cm per pixel. Both the orthophoto and the images used to create the map were taken between 10:00 and 14:00 h on April 21, 2021. Figure 14B shows a close-up of the northern part of the town, where the proposed map indicates a *Sargassum* coverage on the beach between 61% and 80% (orange) and between 81% and 100% (dark red). In the close-up of the orthophoto corresponding to this zone, it is possible to observe a quantity of accumulated *Sargassum* related to the orange and dark red colored pixels on the map. Figure 14C shows a coverage between 41% and 60% (yellow), a coverage between 21% and 40% (light green), and a coverage between 0% and 20% (dark green). The close-up of the orthophoto corresponding to this zone confirms that the beach has a moderate presence of *Sargassum* in the yellow and light green areas and that it is clean in the areas corresponding to the sections colored in dark green. Finally, Fig. 14D shows regions colored in dark green, suggesting a minimum *Sargassum* coverage on the beach of between 0% and 20%, *i.e.,* it represents a relatively clean area of *Sargassum*, as confirmed by the close-up of the orthophoto section corresponding to this area. In summary, as shown in Fig. 14, the diversity of the photo frames, as well as the ranges suggested for the definition of the colors used to identify the estimate of *Sargassum* coverage, minimized the impact that could be caused by the different angles of inclination at the time of taking the photos.

## Conclusions

The study presented a new methodology for monitoring the *Sargassum* along the beach to support the development of work plans that improve the collection and management of this macroalgae. Due to the increase in mobile devices used (*e.g.*, smartphones, tablets) and the constant modernization of the platforms that interconnect them to the Internet, Crowdsourcing was chosen as the mechanism for image collection in this study.

Crowdsourcing encourages the process of social appropriation of science for the construction of multi-scale approaches used during *Sargassum* monitoring. The acquisition of images through Crowdsourcing allowed to reduce the cost of monitoring compared to methods that could use satellite images of very high spatial resolution, even allowing, in case of having enough information, to perform almost real-time monitoring of the beaches. The above allows the proposed methodology to complement remote sensing technologies, opening the possibility of hybrid beach monitoring.

Unlike monitoring techniques based on satellite images, the atmospheric conditions of the study area have a minimal impact on the proposed methodology. Also, a new dataset was generated, composed of 1,000 segmented images containing information on the *Sargassum* accumulated on the beach. This dataset is freely available for other research related to this topic.

On the other hand, the proposed methodology has several aspects to be improved as part of our future work. The first aspect is related to the dependence on the continuous flow of data; Crowdsourcing does not guarantee this, which prevents monitoring in areas where there is no influx of people, like in natural protected areas. Thus, to reduce possible errors and increase the quality of the maps generated, a continuous flow of images is required.

A second aspect related to crowdsourcing for image acquisition is the absence of metadata, which prevents normalizing the images and thus making better comparisons between them. This hinders determining the error in the generated estimates and knowing the bias. Although this aspect could be minimized with multiple observations of the same area, it is still a point to keep in mind. We believe that these limitations open a possibility for new studies that will help improve the results in the future.

## Additional Information and Declarations

### Data Availability

The following information was supplied regarding data availability: The segmented images are available at figshare: Arellano-Verdejo, Javier (2021): Sargassum Segmented Dataset. figshare. Dataset. https://doi.org/10.6084/m9.figshare.16550166.v1.
